# m^7^GDisAI: N7-methylguanosine (m^7^G) sites and diseases associations inference based on heterogeneous network

**DOI:** 10.1186/s12859-021-04007-9

**Published:** 2021-03-24

**Authors:** Jiani Ma, Lin Zhang, Jin Chen, Bowen Song, Chenxuan Zang, Hui Liu

**Affiliations:** 1 Engineering Research Center of Intelligent Control for Underground Space, Ministry of Education, China University of Mining and Technology, Xuzhou, 221116 China; 2 School of Information and Control Engineering, China University of Mining and Technology, Xuzhou, 221116 China; 3 Department of Biological Sciences, AI University Research Center, Xi’an Jiaotong-Liverpool University, Suzhou, 215123 China

**Keywords:** m^7^G site, Heterogeneous network, Matrix decomposition

## Abstract

**Background:**

Recent studies have confirmed that N7-methylguanosine (m^7^G) modification plays an important role in regulating various biological processes and has associations with multiple diseases. Wet-lab experiments are cost and time ineffective for the identification of disease-associated m^7^G sites. To date, tens of thousands of m^7^G sites have been identified by high-throughput sequencing approaches and the information is publicly available in bioinformatics databases, which can be leveraged to predict potential disease-associated m^7^G sites using a computational perspective. Thus, computational methods for m^7^G-disease association prediction are urgently needed, but none are currently available at present.

**Results:**

To fill this gap, we collected association information between m^7^G sites and diseases, genomic information of m^7^G sites, and phenotypic information of diseases from different databases to build an m^7^G-disease association dataset. To infer potential disease-associated m^7^G sites, we then proposed a heterogeneous network-based model, m^7^G Sites and Diseases Associations Inference (m^7^GDisAI) model. m^7^GDisAI predicts the potential disease-associated m^7^G sites by applying a matrix decomposition method on heterogeneous networks which integrate comprehensive similarity information of m^7^G sites and diseases. To evaluate the prediction performance, 10 runs of tenfold cross validation were first conducted, and m^7^GDisAI got the highest AUC of 0.740(± 0.0024). Then global and local leave-one-out cross validation (LOOCV) experiments were implemented to evaluate the model’s accuracy in global and local situations respectively. AUC of 0.769 was achieved in global LOOCV, while 0.635 in local LOOCV. A case study was finally conducted to identify the most promising ovarian cancer-related m^7^G sites for further functional analysis. Gene Ontology (GO) enrichment analysis was performed to explore the complex associations between host gene of m^7^G sites and GO terms. The results showed that m^7^GDisAI identified disease-associated m^7^G sites and their host genes are consistently related to the pathogenesis of ovarian cancer, which may provide some clues for pathogenesis of diseases.

**Conclusion:**

The m^7^GDisAI web server can be accessed at http://180.208.58.66/m7GDisAI/, which provides a user-friendly interface to query disease associated m^7^G. The list of top 20 m^7^G sites predicted to be associted with 177 diseases can be achieved. Furthermore, detailed information about specific m^7^G sites and diseases are also shown.

**Supplementary Information:**

The online version contains supplementary material available at 10.1186/s12859-021-04007-9.

## Introduction

Over 150 types of RNA modifications have been identified in RNA molecules [1, 2], and N7-methylguanosine (m^7^G), which refers to methylation of guanosine(G) on position N7 is a typical positively charged modification present in tRNA [3], rRNA [4], mRNA 5′cap [5] and internal mRNA regions [6], playing a critical role in regulating RNA processing, metabolism,and function. As a positively charged RNA modification, m^7^G could tune RNA secondary structures or protein-RNA interactions through a combination of electrostatic and steric effects [7]. m^7^G sites in several tRNAs variable loops, which are installed by the heterodimers METTL1-WDR4 in mammals [3], have been reported to stabilize tRNA tertiary fold [8, 9]. m^7^G sites that install at 5′cap stabilize transcripts against exonucleolytic degradation [10], and modulate nearly every stage of the mRNA life cycle, including transcription elongation [11], pre-mRNA splicing [12], polyadenylation [13], nuclear export [14], and translation [15].

Mutations in m^7^G methyltransferase are associated with various diseases. To be more specific, a mutation in the methyltransferase complex WDR4 (WD Repeat Domain 4) in humans has been reported to cause primordial dwarfism characterized by facial dysmorphism, brain malformation, and severe encephalopathy with seizures [16, 17]. Lin et al. [18] reported that knockout of the m^7^G46 tRNA WDR4 in embryonic stem cells impairs neural lineage differentiation and affects translation on a global scale. Besides, overexpression of WDR4 has been discovered to influence learning and memory in Down syndrome [19]. Moreover, the m^7^G tRNA methyltransferase METTL1 (Methyltransferase like 1) was reported to influence cancer cell viability [20]. Therefore, identification of disease-associated m^7^G sites will accelerate the understanding of disease pathogenesis at the molecular level, and will further benefit the prognosis, diagnosis, evaluation, treatment, and prevention of human complex diseases. However, it is time-consuming and expensive to explore the association between m^7^G sites and various diseases by only conducting wet experiments. Fortunately, m^7^G-MeRIP-Seq [21], m^7^G-miCLIP-seq [6], and m^7^G-Seq [21] have generated vast amounts of biological data about m^7^G, so computational methods are urgently needed to uncover potential disease-associated m^7^G sites effectively. Researchers can then select the most probable m^7^G sites and the host genes of these sites for further analysis, streamlining their wet-lab experiments. To our knowledge, no computational models for finding disease-associated m^7^G sites have been developed.

In this study, we extracted 768 validated associations among 741 m^7^G sites and 177 diseases from m^7^GHub to construct the m^7^G disease association dataset [22]. Then we proposed a heterogeneous network-based m^7^G-disease associations inference method m^7^GDisAI to prioritize candidate m^7^G sites for a disease of interest. Furthermore, experiments of cross validation and case study on ovarian cancer have been carried out to prove the effectiveness and stability of our method. To facilitate the exploration and direct query of our predicted results, we developed an online database m^7^GDisAI. The website hosts the top 20 m^7^G sites predicted to be associated with 177 diseases with high prediction scores and supports queries with diseases which you are interested. The m^7^GDisAI website is freely available at http://180.208.58.66/m7GDisAI/.

## Implementation

### Datasets

#### Source of datasets

m7GHub is a comprehensive m^7^G online platform, which deciphers the location, regulation, and pathogenesis of m^7^G modification [22]. It consists of four parts, including m7GDB, m7GFinder, m7GSNPer, and m7GdiseaseDB. It provides 69,159 m^7^G sites which are classified into three confidence levels: high confidence level sites reported by m^7^G-seq, medium confidence level sites reported by m^7^G-MeRIP-Seq as well as m^7^G-miCLIP-Seq, and low confidence level sites predicted by m7GFinder. As a subpart of m7GHub, m7GDiseaseDB collects 1218 disease-associated genetic variants that may lead to gain/loss of m^7^G sites, with implications for disease pathogenesis involving m^7^G RNA methylation. It provides us sufficient information to construct the m^7^G-variant dataset and further build the m^7^G-disease association dataset.

#### m^7^G-variant dataset

In the m^7^G-variant dataset, m^7^G-associated variants refer to those mutated at or close to G sites and cause gain/loss of m^7^G sites simultaneously. For each m^7^G site-variant pair, the association of them was measured by the association levels as well as the confidence levels. The association level qualifies the influence that variants exert on m^7^G sites into the range [0,1]. The closer the association level is to 1, the stronger influence that variant exerts on the exact site. Initially, 812 m^7^G site-variant pairs with high confidence level were first extracted, then ranked according to the association level. Then 741 m^7^G site-disease pairs were further picked out with association levels higher than 0.8. Meanwhile, the sequence and genomic location information of m^7^G-variant pairs were collected correspondingly in this dataset. Specifically, it contains the genomic locations, host genes of m^7^G sites, site-centered 41 bp reference sequences as well as site-centered 41 bp alternative sequences.

#### m^7^G-disease association dataset

In the m^7^G-disease association dataset, 741 m^7^G sites were associated with 177 diseases via 741 variants in the m^7^G-variant dataset. Specifically, these variants are both m^7^G-associated and disease-associated. In other words, they cause the gain/loss of the m^7^G site and involve in various disease pathogenesis. Taking these variants as linkages, 177 diseases in ClinVar and GWAS were found to be associated with 741 variants, with implications for disease pathogenesis in m^7^G RNA methylation.

### Methods

**Fig. 1:**
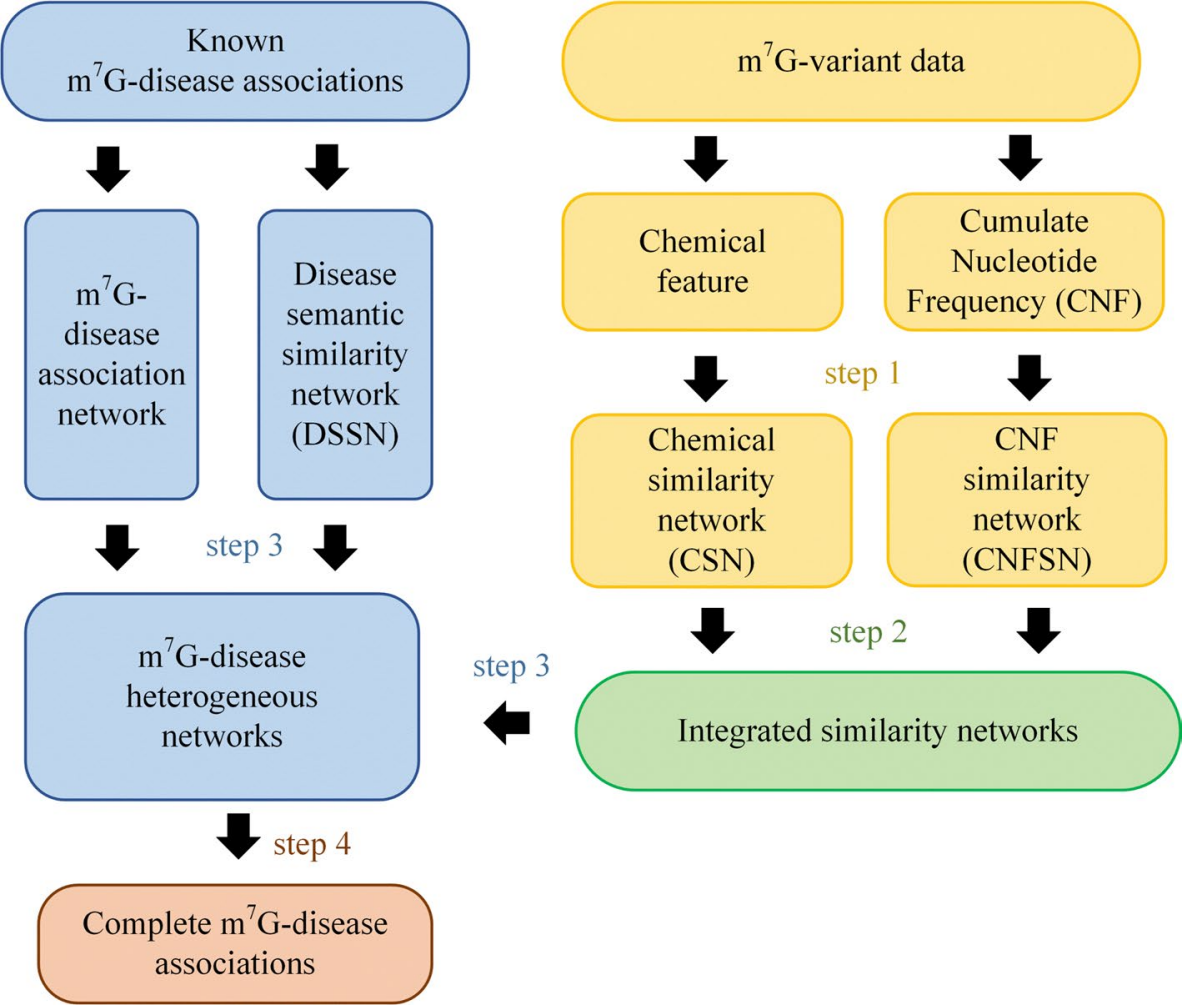
The framework of m^7^GDisAI. m^7^GDisAI mainly consists of four steps. The first step is to extract m^7^G sequence-derived features with m^7^G-variant data to construct m^7^G chemical similarity network (CSN) and CNF similarity network (CNFSN). The second step is to fuse CSN and CNFSN together by taking linear combinations of chemical similarities and CNF similarities, and then form a series of m^7^G integrated similarity networks. The third step is to build heterogeneous networks with m^7^G-similarity networks, m^7^G-disease association network, and disease semantic network. The fourth step is to predict associations between unknown m^7^G site-disease pairs

m^7^G-disease association network reconstruction can be transformed into predicting the unknown entries in the m^7^G-disease association matrix, which can be solved by traditional matrix decomposition methods. However, the number of known associations is so small that matrix decomposition methods cannot achieve satisfactory performance in this case. Thus, we proposed a heterogeneous network-based m^7^G-disease association prediction method m^7^GDisAI which will be detailed in the next. The framework of m^7^GDisAI is shown in Fig. 1.

#### m^7^G-Disease Association Network

Based on the m^7^G-disease association dataset, the m^7^G-disease adjacency network was constructed to record their associations. To be more specific, let *S* = {*s*_*1*_*, s*_*2*_*, …, s*_*m*_} and *D* = {*d*_*1*_*, d*_*2*_*, … d*_*n*_} denote *m* m^7^G sites and *n* diseases respectively. Let ${\varvec{A}}_{{{\varvec{SD}}}} \in R^{m \times n}$ indicate the adjacency network, ${\varvec{A}}_{{{\varvec{SD}}_{{{\mathbf{ij}}}} }}$ is 1 if there exists a validated association between m^7^G-disease pair $(s_{i} ,d_{j} )$. The m^7^G-disease association matrix ***A***_***SD***_ was provided in Additional file 4: Table S4.

#### m^7^G similarity networks

As a kind of auxiliary information, m^7^G similarity information plays a critical role in m^7^G-disease association prediction. To make full advantages of the information of m^7^G sites, a series of m^7^G similarity networks were constructed for further use in the heterogeneous network.

##### m^7^G chemical similarity network

m^7^G chemical similarity network (CSN) depicts the m^7^G similarities in terms of the chemical properties extracted from m^7^G site-centered sequences [23, 24]. Specifically, either sequence is a combination of four nucleotides A, T, C, G. Each nucleotide can be characterized by three distinct structural chemical properties, such as ring structures, hydrogen bonds, and functional groups. In terms of ring structures, A and G have two benzene rings, while C and T have only one. As for the number of hydrogen bonds formed during hybridization, A and T have two, while G and C have three. Regarding the functional groups they contain, A and C contain amino groups, whereas G and T contain keto groups. Therefore, the *i*-th nucleotide in sequence *N* can be encoded by a vector $(x_{i} ,y_{i} ,z_{i} )$.

$$x_{i} = \left\{ {\begin{array}{*{20}c} 1 & {{\text{if }}\;N_{i} \in \{ A,G\} } \\ 0 & {{\text{if }}\;N_{i} \in \{ C,T\} } \\ \end{array} } \right\},\quad y_{i} = \left\{ {\begin{array}{*{20}c} 1 & {{\text{if }}\;N_{i} \in \{ A,T\} } \\ 0 & {{\text{if }}\;N_{i} \in \{ G,C\} } \\ \end{array} } \right\},\quad z_{i} = \left\{ {\begin{array}{*{20}c} 1 & {{\text{if}}\; \, N_{i} \in \{ A,C\} } \\ 0 & {{\text{if}}\; \, N_{i} \in \{ G,T\} } \\ \end{array} } \right\}$$

Therefore, A, C, G, T can be encoded as (1,1,1), (0,0,1), (1,0,0) and (0,1,0) respectively. Thus, the chemical feature of site *s*_*i,*_ denoted as CF (*s*_*i*_), is the combination of these four vectors, in the form of a sequence consisting of {0,1}. Considering the binary numerical properties of the m^7^G chemical features, the Jaccard coefficient was applied to them. To be specific, for two sites *s*_*i*_ and *s*_*j*_, their pairwise chemical similarity is defined as (1)

1 $$che\_sim_{ij} = \frac{{|CF(s_{i} ) \cap CF(s_{j} )|}}{{|CF(s_{i} ) \cup CF(s_{j} )|}}$$

Then in the m^7^G CSN, *s*_1_, *s*_2_, …, *s*_m_ are nodes, and the edges between them are weighted by the pairwise chemical similarity above. For convenience, the adjacency matrix was indicated as ***A***_***CSN***_ (Additional file 5: Table S5).

##### m^7^G Cumulative Nucleotide Frequency Similarity Network

Similar to the construction of CSN, m^7^G cumulative nucleotide frequency (CNF) features were extracted for further similarity calculation. To be specific, CNF of the *i*-th nucleotide in a sequence is defined as the sum of all the instances of this nucleotide before the *i* + 1 position dividing *i*. Taking the sequence ‘TAAGTCCA’ as an example, the CNF for A is 0.5(1/2),0.667(2/3),0.375(3/8) at the 2nd, 3rd and 8th positions respectively. Thus, the CNF features of site *s*_*i*_ are denoted as CNF (*s*_*i*_). Comparing with the m^7^G chemical features, CNF features pay more attention to the sequence context around the m^7^G site. Then the Cosine coefficient was adopted to calculate similarities of CNF since it reflects the similarity in trend rather than absolute values. For sites *s*_*i*_ and *s*_*j*_, the pairwise CNF similarity is defined as (2).

2 $$CNF\_sim_{ij} = \frac{{|CNF(s_{i} ) \cdot CNF(s_{j} )|}}{{||CNF(s_{i} )||_{2} ||CNF(s_{j} )||_{2} }}$$

Then m^7^G CNF similarity network (CNFSN) was obtained with the weights between nodes *s*_*i*_ and *s*_*j,*_ (*i* = 1,2…m, *j* = 1,2…m), and the adjacency matrix was indicated as ***A***_***CNFSN***_ (Additional file 6: Table S6).

##### m^7^G integrated similarity network

Since m^7^G chemical similarity and CNF similarity measure m^7^G similarities from their own views, we took a linear combination of those two similarities to form an integrated similarity, and the contribution of m^7^G chemical similarity and CNF similarity is weighted by *α*. For sites *s*_*i*_ and *s*_*j*_, the integrated similarity is defined as (3).

3 $$int\_sim_{ij} = (1 - \alpha ) \cdot che\_sim_{ij} + \alpha \cdot CNF\_sim_{ij}$$

The value of *α* was chosen from 0 to 1 with step 0.1, and was determined by tenfold cross validation experiments. Then a series of m^7^G integrated similarity networks were obtained via taking (3) as weights between nodes *s*_*i*_ and *s*_*j,*_ (*i* = 1,2…m, *j* = 1,2…m), and its adjacency matrix was indicated as ***A***_***SS***_. In addition, if *α* is 0, then ***A***_***SS***_ is ***A***_***CSN***_, while if *α* is 1, then ***A***_***SS***_ is ***A***_***CNFSN***_.

#### Disease semantic similarity network

Disease semantic similarity network (DSSN), indicated by adjacency matrix ***A***_***DD***_, was also constructed by calculating pairwise disease semantic similarities. Generally speaking, functional similarity between molecules results in similar phenotypes, such as diseases. Based on this fact, many researchers [15, 25–27] utilized functional similarities of the disease-associated molecules for semantic disease similarities. We followed Wang’s PBPA method, which was implemented to calculate pairwise disease semantic similarities [28, 29]. Additionally, the “DisSetSim” web server can be accessed from http://www.bio-annotation.cn:18080/DincRNAClient. By calculating all pairwise semantic similarities in *D,* a disease semantic similarity network was obtained and the adjacency matrix was indicated as ***A***_***DD***_ (Additional file 7: Table S7).

#### m^7^G-disease heterogeneous network

**Fig. 2:**
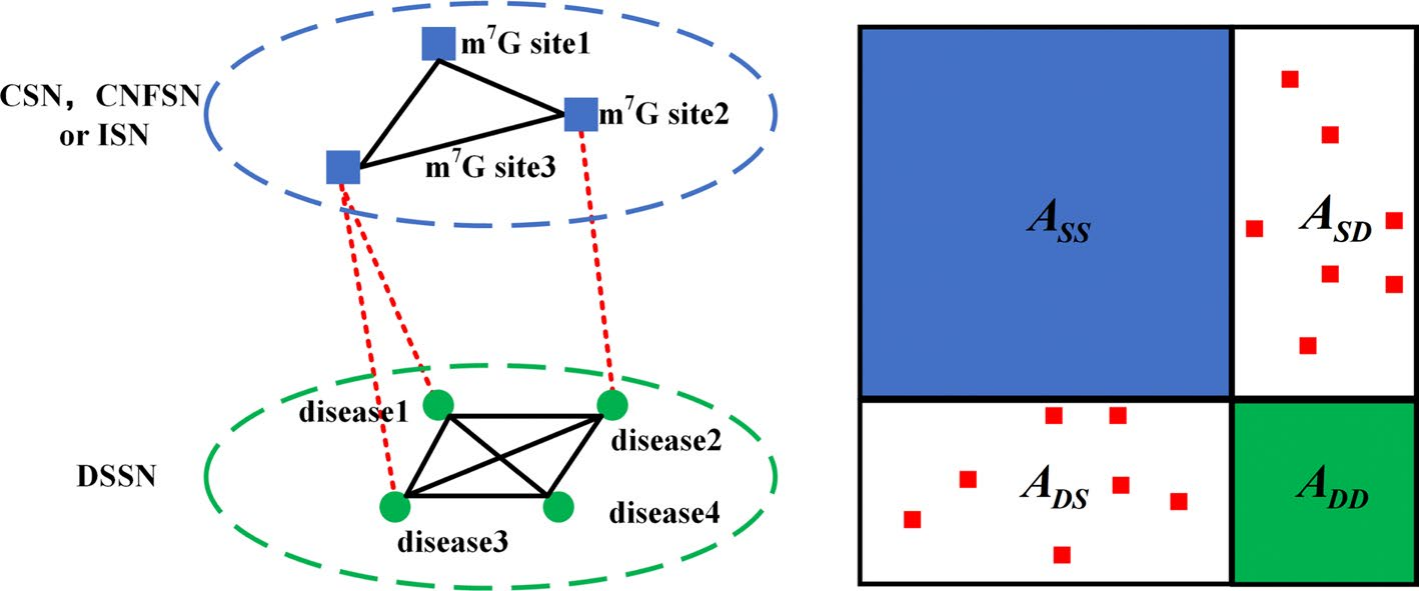
m^7^G-disease heterogeneous network and its adjacency matrix

The m^7^G-disease heterogeneous network and its adjacency matrix are shown in Fig. 2. The m^7^G-disease heterogeneous network was constructed by incorporating m^7^G-disease adjacency network, disease semantic similarity network DSSN, and m^7^G integrated similarity networks. It was represented by adjacency matrix ***A*** and mask matrix ***W***, as (4).

4 $${\varvec{A}} = \left( {\begin{array}{*{20}c} {{\varvec{A}}_{{{\mathbf{SS}}}} } & {{\varvec{A}}_{{{\mathbf{SD}}}} } \\ {{\varvec{A}}_{{{\mathbf{SD}}}}^{{\mathbf{T}}} } & {{\varvec{A}}_{{{\mathbf{DD}}}} } \\ \end{array} } \right),{\varvec{W}} = \left( {\begin{array}{*{20}c} {{\varvec{W}}_{{{\mathbf{SS}}}} } & {{\varvec{W}}_{{{\mathbf{SD}}}} } \\ {{\varvec{W}}_{{{\mathbf{SD}}}}^{{\mathbf{T}}} } & {{\varvec{W}}_{{{\mathbf{DD}}}} } \\ \end{array} } \right)$$

where ***W***_***SS***_ and ***W***_***DD***_ are all one’s matrix. For ***W***_***SD***_, ***W***_***ij***_ = 1 if the association of the *i*-th site to the *j*-th disease is known, 0, vice versa.

By incorporating DSSN and m^7^G integrated similarity networks into the m^7^G-disease adjacency network, cold start issue is avoided, while information of sites and diseases is fully be used.

#### m^7^G-disease association inference based on heterogeneous network

Based on the m^7^G-disease heterogeneous network constructed above, the goal of recovering ***A***_***SD***_ is transformed into completing ***A***. Underpinned by the fact that similar sites have similar molecular pathways for similar diseases, the matrix completion model assumes that the underlying latent factors determining m^7^G-disease associations are highly correlated. In addition, if two sites are similar, then they would have similar patterns with any other sites, and it is true for diseases. The number of independent factors that govern the pattern of ***A*** is much smaller than that of sites and diseases. In a mathematical view, the number of independent factors is the rank, here we used *k* to denote it. Thus, the goal of completing ***A*** can be achieved by the classical matrix decomposition method, which achieved positive results in many cases and is easy to realize. The primary idea of matrix decomposition is to map the adjacency matrix ***A*** into a *k* dimensional space, where *k* <  < *m* + *n*, so dimension reduction is achieved and a lower-dimensional representation of ***A*** in a *k*-dimensional space is given by two matrices ${\mathbf{U}} \in {\mathbb{R}}^{(m + n) \times k}$ and ${\mathbf{V}} \in {\mathbb{R}}^{(m + n) \times k}$. Then ***A*** can be approximated by (5).

5 $${\varvec{A}} \approx {\varvec{UV}}^{{\mathbf{T}}}$$

The fundamental idea of finding suitable factor matrices ***U***, ***V*** is to minimize the objective function defined as (6):

6 $$\mathop {min}\limits_{{{\varvec{U,V}}}} ||{\varvec{W}} \odot ({\varvec{A}} - {\varvec{UV}}^{{\mathbf{T}}} )||_{F}^{2}$$

where $|| * ||_{F}$ is the Frobenius norm, ${\varvec{W}} \odot ({\varvec{A}} - {\varvec{UV}}^{{\mathbf{T}}} )$ denotes the Hadamard product of two matrices ***W ***and ***A-UV***^**T**^.

Furthermore, regularization terms should be considered, and the loss function is defined as (7), while the objective function is (8).

7 $$L = ||{\varvec{W}} \odot \left( {{\varvec{A}} - {\varvec{UV}}^{{\mathbf{T}}} } \right)||_{F}^{2} + \lambda_{{1}} {||}{\varvec{U}}||_{F}^{2} + \lambda_{2} {||}{\varvec{V}}||_{F}^{2}$$

8 $$\mathop {\min }\limits_{{{\varvec{U,V}}}} \, L$$

where $\lambda_{{1}} {||}{\varvec{U}}||_{F}^{2} + \lambda_{2} {||}{\varvec{V}}||_{F}^{2}$ is the regularization term to avoid overfitting, with *λ*_1_ and *λ*_2_ being the regularization parameters.

*λ*_1_ and *λ*_2_, which were optimized by cross validation, help to achieve the trade-off between fitting and generalization. The Alternating Least Square method [30, 31] was then followed to reach the global minimum concerning to ***U*** and ***V***. Finally, unknown entries in ***A***_***SD***_ were predicted. The implementation process of m^7^GDisAI is given below.
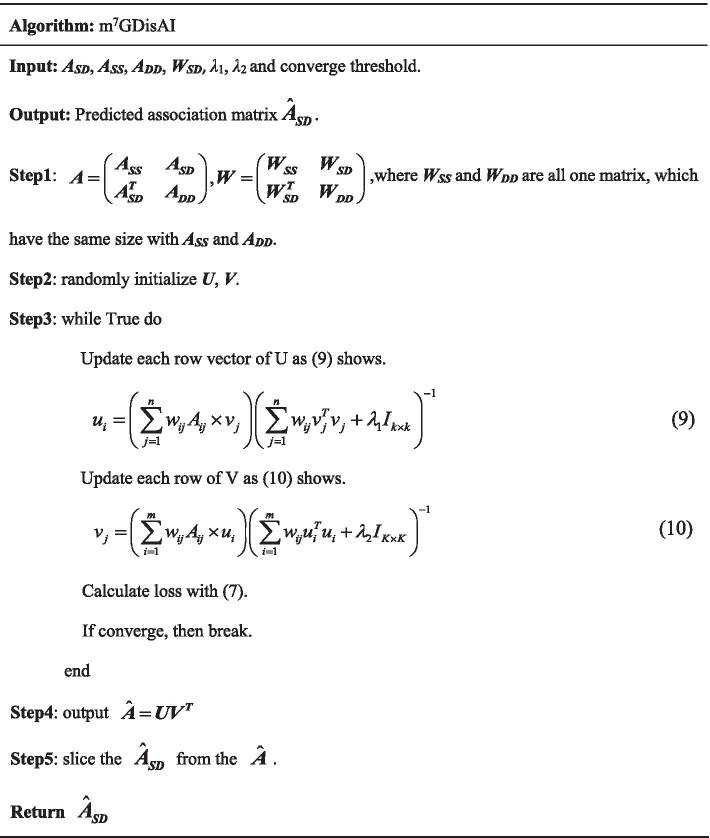


## Results

### Experimental design

To systematically evaluate the prediction performance of m^7^GDisAI on the m^7^G-disease association dataset, tenfold cross validation and LOOCV strategies were adopted for the experiments.

As for tenfold cross validation, in the m^7^G-disease association dataset, there are 768 validated known associations, and the others that haven’t been validated are considered as candidate associations. All known associations are randomly divided into 10 sets that are roughly equal size. Each set is taken as test set in turn, in other words, pretends to be unknown ones, while the remaining nine sets serve as the training set. After performing m^7^GDisAI on training set, the test associations were ranked together with the candidate associations in descending order according to the predicted value obtained by m^7^GDisAI. Additionally, two types of LOOCV, global LOOCV and local LOOCV, were further carried out on the m^7^G-disease association dataset. At each iteration, each validated known m^7^G-disease association was treated as the test data and all the remaining associations as the training data. The only difference between them is the selection of candidate samples. To be specific, in global LOOCV, the candidate samples are all unknown m^7^G-disease associations, while in local LOOCV, candidate samples are only those associations under the disease of interest. In each scheme of LOOCV, the test sample was ranked with candidate samples in descending order.

Regardless of tenfold cross validation, global LOOCV and local LOOCV, for a given threshold *τ*, a test association is regarded as true positive (TP) if it ranks above the threshold, false negative (FN) otherwise. Similarly, a candidate sample is considered as false position (FP) if it ranks above the threshold, true negative (TN) otherwise. By varying *τ*, true positive rate (TPR), false positive rate (FPR) can be calculated for Receiver Operating Characteristic (ROC) curve. It depicts the relative tradeoffs of prediction performance between TP and FP [32]. The area under ROC curve (AUC), ranging from 0 to 1, can be used to evaluate the overall performance [32, 33].

### Parameter setting

There are four parameters, rank *k*, linear combination coefficient *α*, regularization parameters *λ*_1_ and *λ*_2_, that are required to be optimized to enhance the performance of m^7^GDisAI. To be specific, *k* is the number of independent factors that govern the pattern of the heterogeneous matrix ***A***, and if *k* is too large, then the algorithm would be time-consuming. Then *k* is chosen from {70,90,110}. The linear combination coefficient *α* weights the contribution of m^7^G chemical similarity and m^7^G CNF similarity in m^7^G integrated similarity network, and it was taken from 0 to 1.0 with the step 0.1. In addition, regularization parameters *λ*_1_ and *λ*_2_ control the relative penalty extent of the factor matrices ***U*** and ***V*** respectively, and they were chosen from {2^–2^,2^–1^,2^0^,2^1^,2^2^}. It is apparent that *k*, *λ*_1_ and *λ*_2_ directly influence the optimal solution of the two factor matrices ***U*** and ***V***, while α only has an impact on the m^7^G similarity matrix ***A***_***SS***._ Thus, *α* was first fixed to 0.5 or any other specific value between 0 to 1, and a grid search strategy was performed on *k*, *λ*_1_ and *λ*_2_. tenfold cross validation experiments were performed with all combination of *k*, *λ*_1_ and *λ*_2_ on the training set. m^7^GDisAI performed best when *k* is 90, *λ*_1_ is -2 and *λ*_2_ is -2 with AUC of 0. 728. For fairness, the impact of *α* on m^7^GDisAI was measured via tenfold cross validation experiments with fixed *k*, *λ*_1_ and *λ*_2_. To be specific, *α* is 0 means that ***A***_***SS***_ is ***A***_***CHN***_, and m^7^GDisAI only utilizes m^7^G chemical similarities, while *α* is 1 indicates that ***A***_***SS***_ is ***A***_***CNFHN***_, and m^7^GDisAI only utilizes m^7^G CNF similarities. Table 1 reports the AUC scores with all *α*, and the highest AUC score is marked in bold.

**Table 1:** AUC scores of different *α* inthe10-fold cross validation experiments

*α*	0	0.1	0.2	0.3	0.4	0.5	0.6	0.7	0.8	0.9	1.0
AUC	0.700	0.703	0.706	0.722	0.705	0.728	0.731	0.733	0.737	0.740	**0.742**

In Table 1, As *α* increases, AUC scores generally show an increased tendency except when *α* is 0.4, and reaches its maximum at 0.742 when *α* is 1. In other words, the more CNF similarities contribute, the higher the AUC scores achieved, and m^7^GDisAI has the best performance when only utilizes CNFHN. Table 1 validates the effectiveness of the CNF features and Cosine coefficient to some extent. Specifically, chemical features decode the nucleotides of m^7^G site-centered sequence individually, while CNF features pay more attention to the context of site-centered sequence. Meanwhile, the Cosine coefficient reflects the similarity in trend instead of absolute value as the Jaccard coefficient calculates.

### Performance evaluation

**Fig. 3:**
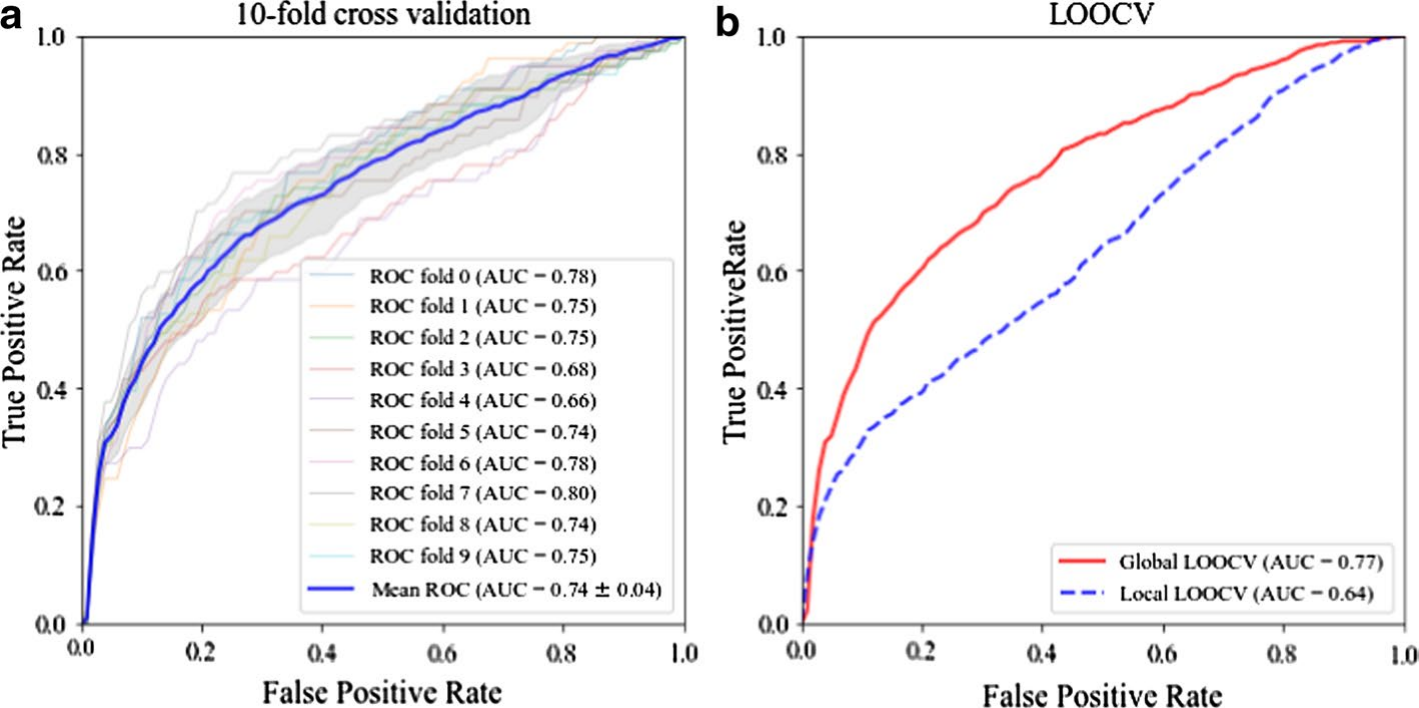
The best performance of m^7^GDisAI for tenfold cross validation and LOOCV experiments. **a.** ROC curves generated by tenfold cross validation. **b.** ROC curves generated by global LOOCV and local LOOCV

To further evaluate the robustness of m^7^GDisAI, we conducted 10 runs of tenfold cross validation experiments by taking *α* as 1, which has the best performance in the Table 1. The mean value of AUC scores is 0.740 with standard variance at 0.0024, showing the effectiveness and stability of m^7^GDisAI. Figure 3a clearly displays the ROC curves with respect to the best performance in tenfold cross validation experiments. Additionally, LOOCV experiments were further conducted to comprehensively evaluate the performance of m^7^GDisAI. The AUC of global LOOCV was 0.769 while that of local LOOCV was 0.635. The ROC curves of LOOCV experiments are illustrated in the Fig. 3b.

As we can see from Fig. 3b, local LOOCV experiment performs worse than global LOOCV. The key factor contributing to this phenomenon is the number of candidate samples that the test sample were ranked with. To be specific, the number of candidate samples participating in global LOOCV is much larger than those involved in the local LOOCV. In other words, the local LOOCV experiments have more rigorous requirements for positive results.

### Case study

Ovarian cancer is the most common cause of gynecological cancer-associated death [34]. Over the past decades, the overall cure rate remains approximately 30% [35]. The reason for low cure rate is the late presentation in most cases. 80% of patients have symptoms, however, these symptoms are shared with many more common gynecological conditions [35]. Given the heterogeneity of this disease, it is necessary to explore the disease pathogenesis at molecular and cellular levels. Then taking all known associations as training samples, while other unknown ones as candidate samples. Since CNFHN has the best performance in the tenfold cross validation experiments, then we performed it on the training samples to score the candidate samples, especially those under ovarian cancer. Furthermore, all the m^7^G sites were ranked in descending order according to their association scores with ovarian cancer, and the top 100 m^7^G sites were selected as potential ovarian cancer-associated sites. 98 host genes of these sites were further mapped out. To predict potential cellular processes and molecular functions that involve m^7^G methylation, we used the R package “clusterProfiler” to analyze and visualize the functional profiles of m^7^G host genes.

**Fig. 4:**
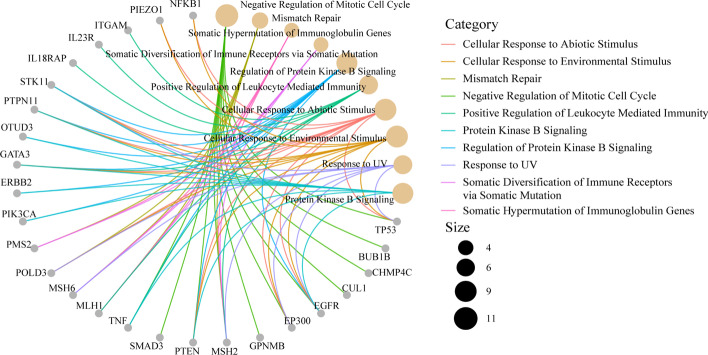
The gene-concept network of functional GO enrichment results. The connection between a gene and a term means that the gene is involved in this GO term

GO terms include three subontologies, cellular component (CC), biological process (BP) and molecular function (MF), and they can be conducted via enrichGO function. In the parameter setting of the enrichGO function, we set the parameter “ont” to “ALL”, aiming at performing CC, BP and MF together. Additionally, the *p*-value cutoff was set as 0.05, *q*-value cutoff 0.2, indicating statistical significance of associations between host genes and GO terms. Furthermore, “BH” method was used to adjust the *p*-value to control the false discovery rate, which was considered to be statistically significant. Considering the potentially biological complexities in which a gene may belong to multiple annotation categories, we utilized a gene-concept network to depict the linkages of gene and GO terms as a network. Figure 4 provides a visualization of the gene-concept network by cnetplot function.

In Fig. 4, ten most significantly enriched terms including CC, BP and MF were shown to be associated with 26 genes. The enrichment analysis results have been verified by published literature. Specifically, TP53 is the most widely studied tumor suppressor gene [36], and it is the host gene of m7G_ID_194615, m7G_ID_203640, m7G_ID_202781 m7G_ID_194736 and m7G_ID_280795 as Additional file 1: Table S1 shows. TP53 functions in ovarian cancer by arresting the cell cycle at G1 phase and by triggering apoptosis [37]. In addition, Lang et al. [38] found that UV radiation leads to base-pair changes of p53, the protein product of the TP53 gene, and further leads to tumor formation. Furthermore, Jeremy et al. [39] experimentally showed that the dynamic patterns of TP53 vary depending on the stimulus. For example, the levels of p53 exhibit a series of pulses with fixed amplitude and frequency in response to DNA breaks caused by γ-irradiation. These discoveries prove that TP53 is enriched into “negative regulation of mitotic cell cycle”, “response to UV” and “cellular response to environmental stimulus” terms [40].

To data, hereditary nonpolyposis colorectal cancer (HNPCC) is the third major cause of hereditary ovarian cancer, and HNPCC is caused by mutations in genes involved in DNA mismatch repair [41]. MLH1 [42] (host gene of m7G_ID_137019, m7G_ID_137020, m7G_ID_151088, m7G_ID_220822), MSH2 [43] (host gene of m7G_ID_161433, m7G_ID_192868, m7G_ID_253317), MSH6 [44] (host gene of m7G_ID_200227, m7G_ID_317794) and PMS2 [45] (host gene of m7G_ID_155289) are all reported to be mismatch repair genes. To be specific, the MLH1 and MSH2 genes are the most common genes for HNPCC-associated ovarian cancer, and account for 80%-90% of observed mutations [46]. What’s more, Cederquist et al. [47] reported that ovarian cancer is in the MSH6 tumor spectrums. Besides, PIK3CA was also known to be oncogenes of ovarian cancer [48], and they are the host genes of m7G_ID_2249, m7G_ID_9238 in Additional file 1: Table S1 respectively. Notably, PIK3CA activated mutation participates in the PI3K pathway which is activated in approximately 70% of ovarian cancer [49], and is enriched in regulation of protein kinase B signaling, which is activated by autocrine or paracrine signaling through protein kinase signaling in many kinds of cancers [49].

Numerical cases [50–52] have suggested that ERBB family of receptor tyrosine kinases has a significant contribution to the initiation and progression of ovarian cancer. EGFR and ERBB2 in Fig. 4 are members of the ERBB family of receptor tyrosine kinases. EGFR is the host gene of m7G_ID_149119 and its overexpression has been observed in 30%-98% of epithelial ovarian cancer in all histologic subtypes, and enhanced expression of EGFR is correlated with advanced-stage disease as well as poor response to chemotherapies. Additionally, Ginath et.al reported [53] that ERBB2 (host gene of m7G_ID_268139) activates multiple downstream signaling pathways, and then promotes the proliferation, invasion, and metastasis of tumor cells.

## Discussion

This research into identifying potential m^7^G-disease association prediction will help us understand the pathogenesis of diseases and promote the treatment of diseases. In this paper, we extracted 768 associations between 741 m^7^G sites and 177 diseases to construct the m^7^G-disease association dataset. To predict the m^7^G-disease association based on the m^7^G-disease dataset, we proposed a heterogeneous network-based association inference method m^7^GDisAI. For m^7^GDisAI, we performed m^7^G-disease association inference on a series of heterogeneous networks which contain m^7^G-disease adjacency network and disease semantic similarity network, but different m^7^G similarity networks, CHN, CNFHN and their combinations.10-fold cross validation, global and local LOOCV were performed with m^7^GDisAI. CNFHN outperforms the CHN and other heterogeneous networks, which proves the effectiveness of CNF features. Then a case study of ovarian cancer was later conducted by CNFHN. It is worth mentioning that the constructed m^7^G-variant pair dataset and m^7^G-disease association dataset may play important role in further investigation of disease-associated m^7^G sites discovery. To our knowledge, m^7^GDisAI is the first algorithm that connects m^7^G sites, variants as well as diseases together to uncover potential cancer-related functions of m^7^G, which may provide some valuable hints for wet experiments guidance. However, there remains limitations in this study. Firstly, the research of m^7^G and diseases is an ongoing topic and the m^7^G-disease dataset is far from completed. Secondly, more feature selection methods could be taken into consideration to construct m^7^G similarity networks and further improve the accuracy of m^7^GDisAI.

## Conclusions

m^7^GDisAI is a heterogeneous network-based m^7^G-disease association inference method and is freely acessible at http://180.208.58.66/m7GDisAI/. m^7^GDisAI uncovers disease-associated m^7^G sites by applying matrix decomposition method on a heterogeneous network-based m^7^G-disease association matrix. m^7^GDisAI provides users a function to query related m^7^G sites of disease which the users are interested in. The website hosts the top 20 m^7^G sites predicted to be associted with 177 diseases with high prediction scores,which may provide some clues for pathogenesis of diseases. The front-end is implemented in JavaScript while the back-end is implemented in Python as well as R. We will continue updating m^7^GDisAI by adding additional information, improving the implementation, and incorporating new measures for infering disease-associated m^7^G sites. The user can always access the latest version of m^7^GDisAI.

## Availability and requirements

Project name: m^7^GDisAI. Project home page: http://180.208.58.66/m7GDisAI/. Operating system(s): Linux, Windows. Programming language: Python, R, JavaScript. Other requirements: Not specified. Python version 3.8.0 or higher, R version 4.0.3 or higher. License: GNU GPL. Any restrictions to use by non-academics: None.

## Supplementary Information

**Additional file 1**.** Table S1**: m7G-variant dataset.

**Additional file 2**. ** Table S2**: The detailed information of diseases we collected.

**Additional file 3**. ** Table S3**: m7G-disease association dataset.

**Additional file 4**. ** Table S4**: m7G-disease association matrix ASD.

**Additional file 5**. ** Table S5**: m7G chemical similarity matrix ACSN.

**Additional file 6**. ** Table S6**: m7G CNF similarity matrix ACNFSN.

**Additional file 7**. ** Table S7**: Disease semantic similarity network ADD.

**Additional file 8**. ** Table S8**: Predicted ovarian cancer related m7G sites and their host genes.

## Data Availability

The detailed information of m^7^G-variant dataset is listed in Additional file 1: Table S1. For each m^7^G-disease pair, information for their sequence and genomic location is included. Additional file 2: Table S2 shows diseases we collected with their names and DOID. Additional file 3: Table S3 provides the information for m^7^G-disease association dataset with 768 known m7G-disease associations. In addition, Additional file 4: Table S4 is the m^7^G-disease matrix *A*_*SD*_ where the validated associations are all one. Additional files 5: Table S5–Additional file 6: Table S6 are m^7^G simialrity networks *A*_*CSN*_, *A*_*CNFSN*_ respectively, while Additional file 7: Table S7 is the disease semantic similarity network *A*_*DD*_. Furthermore, Additional file 8: Table S8 presents the recommended m^7^G sites and their host gene of ovarian cancer. The website m^7^GDisAI implemented to query related m^7^G sites of the disease which you are interested in is deposited at http://180.208.58.66/m7GDisAI/.

## Abbreviations

m^7^G: N7-methylguanosine
m^7^G-MeRIP-Seq: N7-methylguanosine Methylated RNA immunoprecipitation sequencing
m^7^G-miCLIP-Seq: N7-methylguanosineIndividual-Nucleotide-Resolution Crosslinking and Immunoprecipitation
m^7^GDisAI: N7-methylguanosine-disease association inference
CHN: Chemical Heterogeneous Network
CNF: Cumulative Nucleotide Frequency
CNFHN: Cumulative Nucleotide Frequency Heterogeneous Network
LOOCV: Leave-one-out cross validation
DSSN: Disease Semantic Similarity Network
CSN: Chemical Similarity Network
CNFSN: Cumulative Nucleotide Frequency Similarity Network
ISN: Integrated Similarity Network
MICA: Most Informative Common Ancestor
ALS: Alternating Least Squares
FP: False Positive
TN: True Negative
FN: False Negative
ROC: Receiver Operating Characteristic Curves
